# Decomposing geographical judgments into spatial, temporal and linguistic components

**DOI:** 10.1007/s00426-024-01980-7

**Published:** 2024-06-05

**Authors:** Daniele Gatti, Giorgia Anceresi, Marco Marelli, Tomaso Vecchi, Luca Rinaldi

**Affiliations:** 1 Department of Brain and Behavioral Sciences, University of Pavia, Piazza Botta 6, 27100 Pavia, Italy; 2 Department of Psychology, University of Milano-Bicocca, Milan, Italy; 3 NeuroMI, Milan Center for Neuroscience, Milan, Italy; 4 Cognitive Psychology Unit, IRCCS Mondino Foundation, Pavia, Italy

## Abstract

When mentally exploring maps representing large-scale environments (e.g., countries or continents), humans are assumed to mainly rely on spatial information derived from direct perceptual experience (e.g., prior visual experience with the geographical map itself). In the present study, we rather tested whether also temporal and linguistic information could account for the way humans explore and ultimately represent this type of maps. We quantified temporal distance as the minimum time needed to travel by train across Italian cities, while linguistic distance was retrieved from natural language through cognitively plausible AI models based on non-spatial associative learning mechanisms (i.e., distributional semantic models). In a first experiment, we show that temporal and linguistic distances capture with high-confidence real geographical distances. Next, in a second behavioral experiment, we show that linguistic information can account for human performance over and above real spatial information (which plays the major role in explaining participants’ performance) in a task in which participants have to judge the distance between cities (while temporal information was found to be not relevant). These findings indicate that, when exploring maps representing large-scale environments, humans do take advantage of both perceptual and linguistic information, suggesting in turn that the formation of cognitive maps possibly relies on a strict interplay between spatial and non-spatial learning principles.

## Introduction

It is widely assumed that humans primarily rely on specialized spatial mechanisms for the development and organization of mental representations (commonly defined as ‘cognitive maps’) of the physical environment they navigate (O’Keefe & Nadel, 1978a, 1978b; Tolman, 1948). The discovery of neurons selectively tuned to encode spatial information, such as place and grid cells within the hippocampal-entorhinal region, has provided evidence for a fairly specific and mechanistic-detailed neural basis for such cognitive maps (O’Keefe & Dostrovsky, 1971; O’Keefe & Nadel, 1978a, 1978b; Derdikman & Moser, 2010). Recent advancements in the field have further bolstered this view, suggesting that the same neural system involved in spatial navigation would also support the development and organization of non-spatial conceptual knowledge (Bellmund et al., 2018; Bottini & Doeller, 2020a; Stoewer et al., 2022). These perspectives thus prioritize the perceptual and motor origin of cognitive maps, aligning with embodied accounts of cognition (Barsalou, 2008; Gallese & Lakoff, 2005) and positing dependence of mental representations on the re-activation of sensorimotor circuits involved in spatial navigation through simulation processes (e.g., Bottini & Doeller, 2020a).

However, evidence challenging the view of cognitive maps development as primarily relying on specialized spatial computations has also been provided (Louwerse, 2009; Louwerse, 2018; Friedman & Brown, 2000; Friedman & Montello, 2006; Friedman et al., 2002; Gatti et al., 2022; Shrager et al., 2008). For instance, research investigating the origins of biases in humans’ geographical judgments suggest that different sources of information contribute to the organization of spatial knowledge and that, when making spatial judgments, individuals primarily rely on inferences through plausible reasoning (Friedman & Brown, 2000; Friedman & Montello, 2006; Friedman et al., 2002). These inferences would involve propositional and symbolic representations, which are non-spatial in nature. In other words, non-spatial processes would also serve as a key source of biases and distortions in humans’ spatial representations. In addition to this, Shrager and colleagues (2008) demonstrated that patients with hippocampal and entorhinal cortex lesions were able to accurately keep track of reference locations and estimate distances similarly to healthy controls. Thereby acknowledging that this study employed short paths allowing for retaining in working memory (Shrager et al., 2008), this evidence still contrasts the notion of spatial computations relying on hippocampal and entorhinal regions as the only neural processes responsible for the development of mental representation of the environment.

This view is substantiated by a growing body of research emphasizing the crucial interplay between perceptual and linguistic experiences in the formation of cognitive maps. In particular, studies exploiting distributional semantic models (DSMs) demonstrated that spatial information can be bootstrapped from the statistical structure of natural language (Avery et al., 2021; Gatti et al., 2022; Louwerse, 2018; Rinaldi & Marelli, 2020). For instance, by exploiting Latent Semantic Analysis (Landauer & Dumais, 1997), a traditional count-based DSMs whose first processing step builds on the computation of word co-occurrences, Louwerse and colleagues showed that it is possible to reconstruct the spatial layout (e.g., geographical distance between cities) of maps representing large-scale environments from various real world regions using text corpora written in the corresponding languages (e.g., using American corpora to reproduce the maps of the USA; Louwerse & Zwaan, 2009; Louwerse et al., 2012), as well as fictional words such as the Middle Earth from *J. R. R. Tolkien*’s novels (Louwerse & Benesh, 2012). Besides computational evidence, previous studies additionally showed that linguistic components (as estimated from raw co-occurrence frequency of word pairs extracted from a large text corpus, Tillman et al., 2013; or as extracted from DSMs, Gatti et al., 2022) can also account for humans’ performance in spatial tasks. In particular, Tillman and colleagues (2013) presented participants with US cities pairs in their iconic (i.e., northern city above southern city) or reverse-iconic positions (i.e., southern city above northern city) and required them to indicate whether they were closely located or not. By examining response times, their results revealed that linguistic factors (as indexed by the frequency of word co-occurrence) influenced response latencies only in the reverse-iconic order (Tilman et al., 2013). This suggests that when perceptual simulation becomes more challenging, individuals may rely on alternative sources of information and strategies, such as non-spatial processes based on language statistics. However, whether linguistic factors more generally account for human performance when producing distance estimates (i.e., in a spatial task not specifically relying on specific iconic or reverse-iconic spatial arrangements, allowing for a perceptually independent exploration of the influence of linguistic experience on humans’ cognitive maps) is still unknown.

In addition to the role of perceptual and linguistic experience outlined above, temporal information, such as the time required to travel between different locations, might be involved as well when representing spatial layouts. This phenomenon would be due to the interconnected nature of the concepts of space and time at both the cognitive and neural level (MacDonald et al., 2011; Jaszczolt, 2012; Pastalkova et al., 2008; Riemer et al., 2018). Since spatially closer locations can require longer travel time than more spatially distant locations, and vice versa, investigating whether spatial representations can be bootstrapped from temporal information is an important research question warranting further exploration. Critically, studies examining the interplay between spatial and temporal processing have primarily focused on (relatively) small to medium environments (i.e., ranging from rooms to cities; e.g., Giraudo & Péruch, 1988; Herman, et al., 1983, 1984; Jansen-Osmann & Berendt, 2005; Kang et al., 2003; Riemer et al., 2014; Riemer et al., 2018; Sadalla & Staplin, 1980), while research at a large level (e.g., countries or continents) has been relatively neglected (but cfr: Maki, 1981; Säisä et al., 1986; Tversky & Schiano, 1989).

In the present study we thus built on this set of evidence as, to date, a comprehensive understanding of the interplay between spatial, linguistic, and temporal information in shaping mental representation of large environments is missing. In particular, we aimed at further shedding light on the factors subserving the processing of distance-like information from cognitive maps, by applying more modern prediction-based DSMs (e.g., Skip-gram and Continuous Bag of Words; Mikolov Chen et al., ) to quantify the role of linguistic experience. These recent models, indeed, rely on cognitively-plausible, associative learning mechanisms and can be thus conceived as computationally-implemented theoretical frameworks of the human semantic memory (Mandera et al., 2017; Günther et al., 2019; Jones et al., 2015). These models serve as a valuable proxy to quantify the role of linguistic experience in shaping knowledge, as they capture meanings from statistical patterns of word distributions in natural language (Lenci, 2018), without directly accessing nor computing any spatial relationships. Interestingly, there is evidence that these models can capture the layout of medium spatial environments (Anceresi et al., 2023) as well as humans’ behavior in the geographical domains (Gatti et al., 2022) and beyond, like in the case of the human body (Gatti et al., 2023).

However, although an increasing body of research emphasizes the significance of non-spatial processes in the development of cognitive maps (Rinaldi & Marelli, 2020), the interplay between spatial and non-spatial processes in the function at hand is still not clear. These unsolved issues were tested across two experiments. First, we conducted a study to explore the relationship between spatial, linguistic and temporal distances. To do so, we exploited DSMs to retrieve linguistic distances, while we quantified temporal distances as the minimum time needed to travel by train between Italian cities, by selecting the main railway stations of Milan (Experiment 1A) and Rome (Experiment 1B) as departing point. Then, we conducted a second behavioral study to investigate whether linguistic and temporal information can account for human performance over and above spatial information in a task in which participants are required to produce distance estimates between cities.

## Experiment 1A

### Methods

#### Stimuli

Sixty-seven Italian cities were selected as stimuli. All the cities were capital of an Italian district (i.e., *Capoluogo di Provincia*). The cities were selected by indicating Milano Centrale (i.e., the central railway station of Milan) as departing point on the *Bahn-Guru* website (https://direkt.bahn.guru/) and retrieving from it all the Italian cities available that were capital of a district. The *Bahn-Guru* resource uses a (legacy) API by Deutsche Bahn to find all direct trains running via a given station within the next 1–2 weeks and it is an open-source software provided by the OK Lab Berlin (https://codefor.de/berlin/). In particular, for each city, from the *Bahn-Guru* website we retrieved an estimate of the minimum time required to travel by train from Milan. Additionally, for each city we also retrieved its geographical coordinates (from: https://github.com/MatteoHenryChinaski/Comuni-Italiani-2018-Sql-Json-excel) and its vector representation from a distributional semantic model (see below).

#### Distributional semantic model

The DSM used here was *fastText* (Bojanowski et al., 2017; Grave et al., 2018). The model was trained on Common Crawl and Wikipedia (around 11 billion words) using the Continuous Bag of Words (CBOW) method, an approach originally proposed by Mikolov et al. (2013a), with position-weights across 300 dimensions, with character n-grams of length 5 and a window of size 5. When using CBOW, the obtained vector dimensions capture the extent to which a target word is reliably predicted by the contexts in which it appears.

With respect to traditional distributional models, whose ability to generate high quality distributed semantic representations is limited to words that are sufficiently frequent in the input data, *fastText* is based on the idea (originally proposed by Schutze, 1993; and realized by Bojanowski et al., 2017) to take into account sub-lexical information by computing word vectors as the sum of the semantic vectors for the character n-grams embedded in each word.

From the Italian semantic space, we thus extracted the vector representations for the names of the 67 cities included and, in addition, the vector of “*Milano*” (Milan) which we used as reference point.

#### Computation of spatial, temporal and language-based distances

Spatial distances (henceforth SpaDist) were computed on the basis of the cities’ geographical coordinates. Specifically, after retrieving longitude and latitude for each city, using the *geosphere* R package (Hijmans, 2022), we computed the distance between the location of each city and the location of Milan.

Temporal distances (henceforth TempDist) were retrieved from the *Bahn-Guru* website (https://direkt.bahn.guru/; see above). This resource provides an estimate of the minimum time of travel by train between two stations.

Language-based distances (henceforth LingDist) between Milan and each city *k* was obtained with the following formula:

$$LingDist= 1-cos\left(\overrightarrow{k}, \overrightarrow{Milano}\right)$$

That is, language-based distances were computed as the cosine of the angle between the vector of each city and the vector of the word “*Milano*” (Milan) as subtracted from 1 to transform them to a distance scale from a proximity scale (i.e., the lower the value the closer the vectors). Language-based distances were computed using the *dist* function of the *proxy* R package (Meyer & Buchta, 2021). The cosine is generally taken as a reliable measure of (linguistic) similarity between vectors (Günther et al., 2019). This measure indexes how similar—in the corpus used in the training phase- is the use of the two words represented by the two vectors included. Consider, for example, the cosine of the angles formed by the vector of the word “Milano” (Milan) and by the vectors of the words “Roma” (Rome), “Taranto” and “Bergamo”. We can observe that the cosine between “Milano” and “Roma” is higher (0.638) as compared with the one between “Milano” and “Taranto” (0.458), thus indicating a higher linguistic similarity. Interestingly, the cosine between “Milano” and “Roma” is higher (0.638) even as compared with the one between “Milano” and “Bergamo” (0.633), which are geographically closer. This example illustrates how this measure can capture geographical information [i.e., cos(Milano,Bergamo) > cos(Milano,Taranto)] but also clarifies that it can additionally index other components (e.g., Milan and Rome are two big Italian cities, as compared with Taranto and Bergano, and are likely used in the language in a similar way).

#### Data analysis and results

**Fig. 1:**
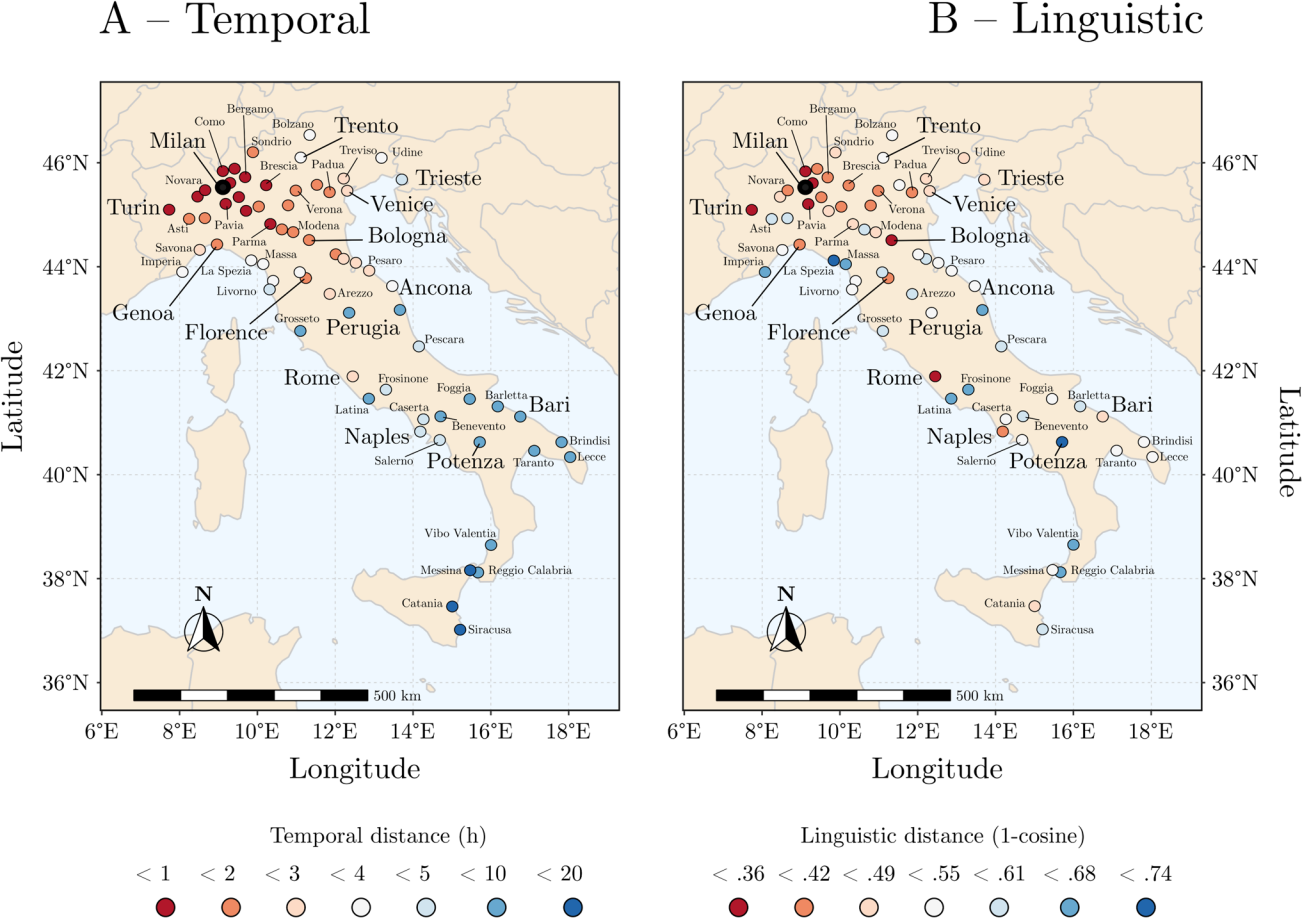
Temporal (**A**) and linguistic (**B**) distance maps from the city of Milan. Warmer colors indicate closer distances. Both temporal and linguistic distances approximate spatial distances

**Fig. 2:**
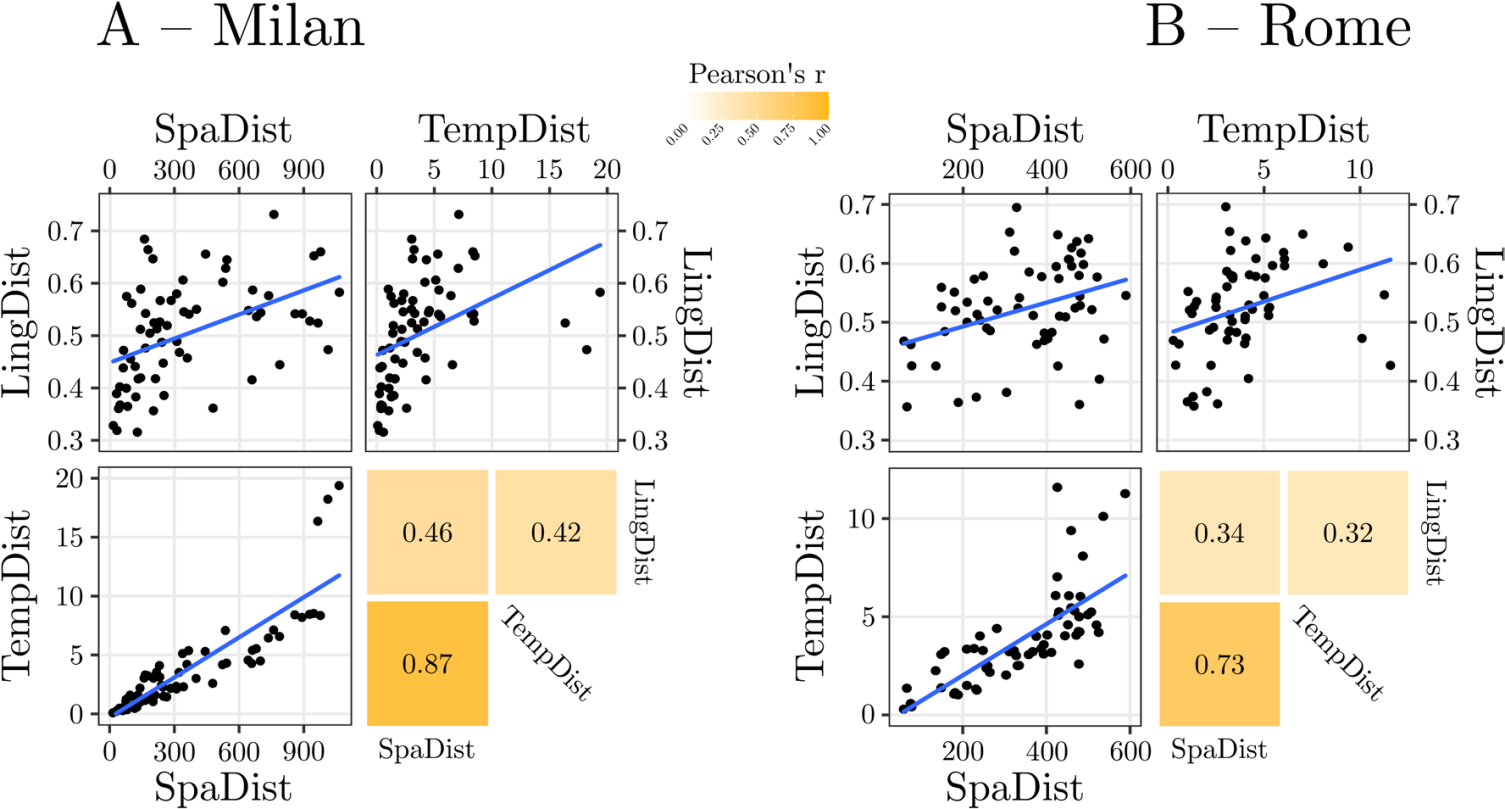
Results of Experiment 1A (**A**) and Experiment 1B (**B**). The two panels report the three scatterplots of the correlations tested and the relative heatmaps (note that the layout of the heatmaps mimic the layout of the scatterplots)

All data, scripts and codes used in the analysis are available at: https://osf.io/pexqm/. All the analyses were performed with *R-Studio* (Rstudio Team, 2015). To investigate the relationship between SpaDist, TempDist and LingDist, we computed a Pearson correlation coefficient matrix. All the correlations were significant (all *p*s < 0.001), specifically the correlation between SpaDist and TempDist had *r* = 0.87, the correlation between SpaDist and LingDist had *r* = 0.46, and the correlation between TempDist and LingDist had *r* = 0.42 (Fig. 1 and Fig. 2A).

## Experiment 1B

### Methods

In Experiment 1B we aimed to replicate the results of Experiment 1A taking “*Roma*” (Rome) as reference point for the computation of both the linguistic and temporal index. The only differences between Experiment 1B and Experiment 1A, thus, are related to the fact the starting cities (i.e., sixty-two Italian cities) were retrieved from *Bahn-Guru* starting from Roma Termini (central Roman station).

#### Data analysis and results

Replicating Experiment 1A, all the correlations were significant (all *p*s < 0.001), specifically the correlation between SpaDist and TempDist had *r* = 0.73, the correlation between SpaDist and LingDist had *r* = 0.34, and the correlation between TempDist and LingDist had *r* = 0.32 (Fig. 2B).

## Experiment 2

Results of Experiment 1 showed significant correlations between spatial, linguistic and temporal distances, highlighting their interconnected nature. However, it remains to be explored whether such linguistic and temporal information can account for human performance in spatial task over and above spatial information. That is, while spatial distances can be in principle retrieved from linguistic and temporal data, whether these two sources of information (language and time) could account for biases in spatial tasks is an open issue. Thus, to test for this, in Experiment 2, we conducted a behavioral task in which participants were required to provide distance judgements between cities. Importantly, since a strong correlation emerged between spatial and temporal distances in Experiment 1, stimuli for Experiment 2 were specifically selected among cities that minimized such correlation for issues of multicollinearity.

### Methods

#### Participants

Fifty-eight students participated in this experiment for course credits (14 males, *M* age = 22.72 years, *SD* = 2.71). All participants were native Italian speakers, had normal or corrected to normal vision and were naïve to the purpose of the study. Informed consent was obtained from all participants before the experiment. The protocol was approved by the psychological ethical committee of the University of Pavia and participants were treated in accordance with the Declaration of Helsinki.

Sample size was determined a-priori based on Brysbaert and Stevens (2018) indication that, in order to achieve properly power, an experiment should have at least 1600 observations per cell of the design (i.e., per condition tested), thus at least 40 stimuli for 40 participants. In our specific case, because we included three continuous predictors in the estimated models, in order to achieve a good level of statistical power the experiment should have had at least 4800 observations. The analysis on accuracy had 7090 observations, while the analysis on correct response latencies had 5984 observations.

#### Stimuli and procedure

Stimuli were selected starting from the cities tested in Experiment 1A. Specifically, starting from the 67 cities included above, we chose 12 cities that were well distributed along the North–South axis and, additionally, those that minimized the correlation between SpaDist and TempDist (i.e., by selecting the ones with the higher error, that is, the ones further from the regression line; see below Computation of spatial, temporal and language-based predictors). The selected cities were: Bologna, Bolzano, Foggia, Grosseto, Latina, Livorno, Napoli, Perugia, Pescara, Rimini, Roma, Taranto. Then, cities were paired to each other, for a total of 132 pairs.

Participants were told that they would have been shown the names of two Italian cities and that they had to indicate which one was geographically closer to Milan. Participants were instructed to respond as fast and accurately as possible by pressing the left/right key (A and L), in order to indicate the city placed on the left side or the one on the right side as being the closer to Milan. The trials were shown in random order.

Each trial started with a central fixation cross (presented for 500 ms) followed by a city-pair (with each city being in either half of the screen; presented for a maximum of 5000 ms) and then, after participants’ response or after 5000 ms, a black screen was presented for 500 ms, which ended the trial. Participants’ responses were recorded only during the 5000-ms word-pair presentation.

Participants were tested online using Psychopy (Peirce, ; Peirce et al., 2019) through the online platform Pavlovia: https://pavlovia.org/.

#### Computation of spatial, temporal and language-based predictors

SpaDist, TempDist and LingDist were retrieved as in Experiment 1A. Then, for each city pair we computed three different predictors, namely a spatial predictor, a temporal predictor and a language-based predictor. The spatial predictor (ΔSpaDist) was computed as the absolute value of the difference between the SpaDists of the two cities comprising the pair. Therefore, the higher the value, the higher the difference (in terms of spatial distance) between the two cities with respect to Milan (i.e., with one city being much closer to Milan compared to the other city in the pair). Similarly, the temporal predictor (ΔTempDist) was computed as the absolute value of the difference between the TempDists of the two cities comprising the pair. Finally, the language-based predictor (ΔLingDist) was computed as the absolute value of the difference between the LingDists of the two cities comprising the pair. The three predictors were retrieved from experiment 1A as to avoid multicollinearity issues. Specifically, they showed small to moderate correlations:* r* = 0.40 for the correlation between ΔSpaDist and ΔTempDist; *r* = − 0.18 for the correlation between ΔSpaDist and ΔLingDists; *r* = 0.26 for the correlation between ΔTempDist and ΔLingDists.

#### Data analysis

All data, scripts and codes used in the analysis are available at: https://osf.io/pexqm/. All the analyses were performed using *R-Studio* (RStudio Team, 2015). Linear mixed models (LMMs) and generalized linear mixed models (GLMMs) were run using the *lme4* R package (Bates et al., 2015). Our dependent variables were participants’ correct response latencies (RTs), which were analyzed using LMMs, and participants’ accuracy, which was analyzed using GLMMs fitted on a binomial family distribution (i.e., correct answers were computed as 1 s and wrong answers as 0 s). To obtain Gaussian data distributions, RTs were log-transformed as their distribution was positively skewed. Marginal *Pseudo-R*^*2*^s are reported.

For the models estimated across both RTs and accuracy, the same three predictors were included in the respective analyses. Specifically, each model had participants and items as random intercepts and, additively, ΔSpaDist, ΔTempDist, and ΔLingDists as continuous predictors (all the three predictors were included in the models as scaled). To further check for multicollinearity, on both models estimated we inspected the variance inflation factor (VIF) of each predictor. This index has 1 as lower boundary indicating no collinearity and has no upper boundary, with its interpretation being: the higher the value, the higher the collinearity. Previous studies discussed various possible thresholds, ranging from 10 (Vittinghoff, 2005), 5 (Menard, 2001), or 2.5 (Johnston et al., 2018). Here, we adopted the latter one, that is the more conservative one.

Finally, to exclude the impact of overly influential outliers, after having fitted the model on RTs, data points were removed on the basis of a threshold of 2.5 *SD* standardized residual errors (model criticism; Baayen, 2008). Results based on the refitted models are reported.

## Results

Trials in which overall RTs were faster than 300 ms or in which participants did not provide an answer (2% of the trials) were excluded from the analyses. Participants correctly answered to 84% of the trials (SD = 5%) and their mean correct response latencies was 1445 ms (SD = 263 ms).

**Fig. 3:**
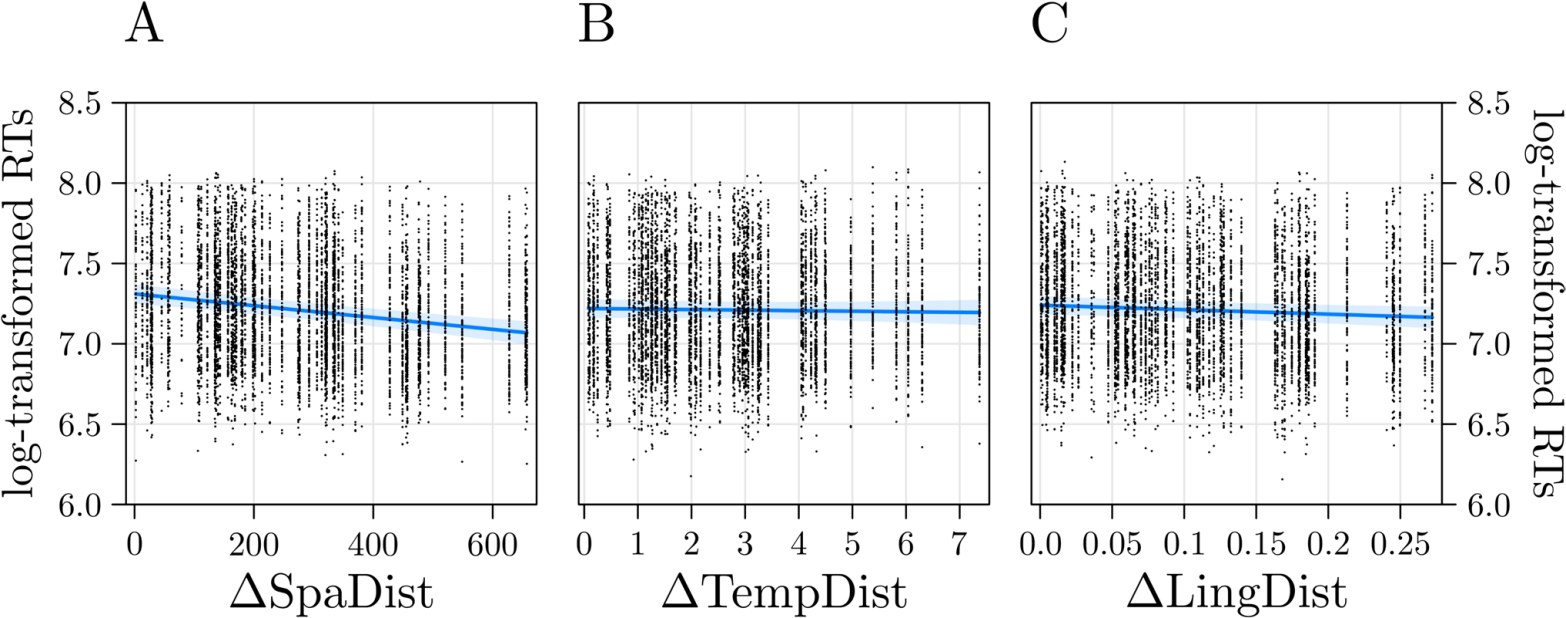
Results of the model estimated on RTs in Experiment 2. Participants’ RTs were predicted by the spatial predictor (**A**), but not by the temporal one (**B**). Interestingly, also the linguistic predictor (**C**) significantly predicted participants’ performance

The model criticism on the model estimated on RTs further removed 77 trials (1.3% of the trials): note that the results reported are fully compatible with the results obtained before model criticism. The model had *Pseudo-R*^*2*^ (marginal) = 0.03 and it showed that the effect of ΔSpaDist was significant, *t*(61.41) =  − 5.97, *p* < 0.001, *b* = − 0.06, *β* = − 0.17, indicating that the higher the ΔSpaDist (the higher the difference between the two spatial distances; i.e., the more distinctively a city of the pair was geographically closer to Milan than the other), the lower participants’ RTs (Fig. 3A). The effect of ΔTempDist was not significant, *t*(59.39) =  − 0.54, *p* = 0.58, *b* = − 0.006, *β* = − 0.01 (Fig. 3B). Interestingly, the effect of ΔLingDist was significant, *t*(61.84) =  − 2.15, *p* = 0.03, *b* = − 0.02, *β* = − 0.06, indicating that the higher the ΔLingDist (the higher the difference between the two linguistic distances; i.e., the more distinctively a city of the pair was linguistically closer to Milan than the other), the lower participants’ RTs (Fig. 3C). All the VIFs were < 1.4, thus excluding multicollinearity issues.

**Fig. 4:**
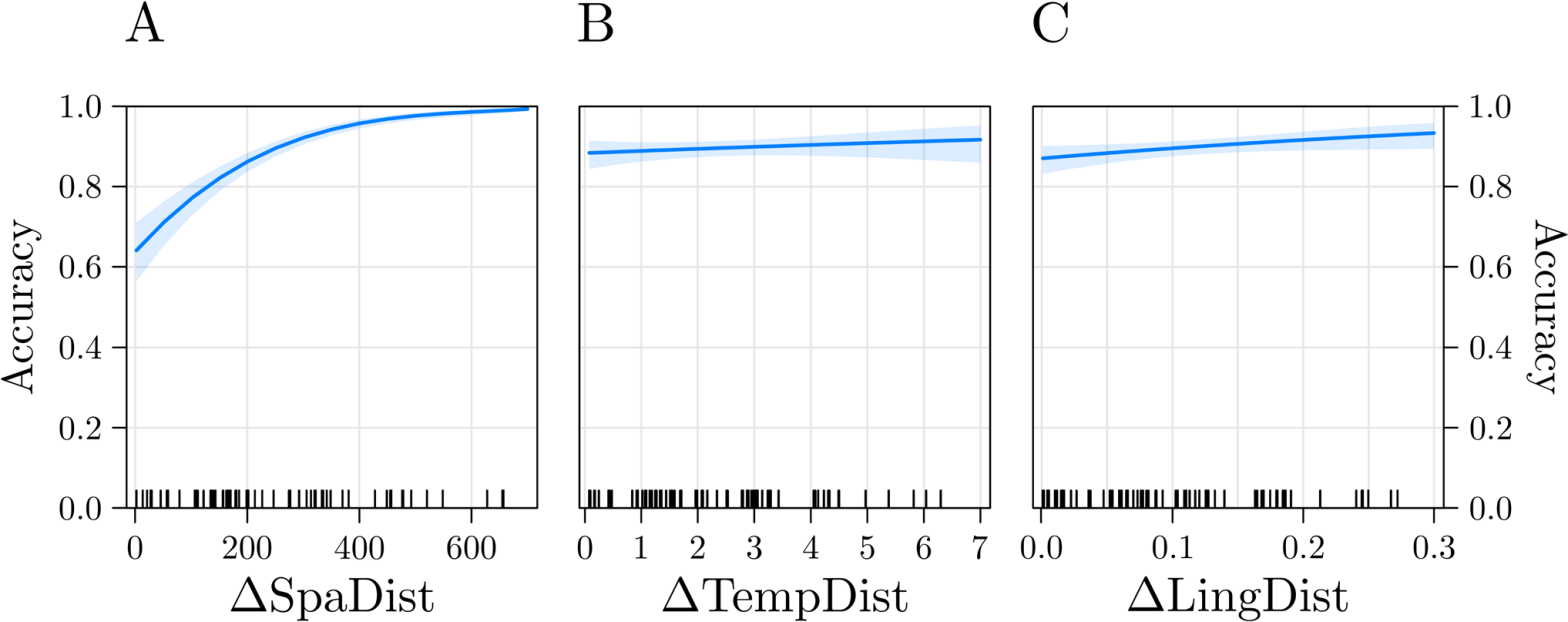
Results of the model estimated on accuracy in Experiment 2. Participants’ accuracy was predicted by the spatial predictor (**A**), but not by the temporal one (**B**). Interestingly, also the linguistic predictor (**C**) significantly predicted participants’ performance

The model estimated on accuracy data had *Pseudo-R*^*2*^ (marginal) = 0.24 and it showed that the effect of ΔSpaDist was significant, *z* = 10.61, *p* < 0.001, *b* = 1.05, indicating that the higher the ΔSpaDist (the higher the difference between the two spatial distances; i.e., the more distinctively a city of the pair was geographically closer to Milan than the other), the higher participants’ accuracy (Fig. 4A). The effect of ΔTempDist was not significant, *z* = 0.88, *p* = 0.38, *b* = 0.09 (Fig. 4B). Interestingly, the effect of ΔLingDist was significant, *z* = 2.06, *p* = 0.03, *b* = 0.18, indicating that the higher the ΔLingDist (the higher the difference between the two linguistic distances; i.e., the more distinctively a city of the pair was linguistically closer to Milan than the other), the higher participants’ accuracy (Fig. 4C). These results indicate that participants relied on both spatial and linguistic knowledge to solve the task. All the VIFs were < 1.25, thus excluding multicollinearity issues.

## Discussion

In the present study, we investigated the interplay between spatial, linguistic, and temporal information in shaping the mental representation of maps representing large-scale environments (e.g., countries or continents). In particular, we addressed this issue by conducting two experiments. In Experiment 1, we employed a computational approach to explore the relationship between spatial, linguistic and temporal distances. To accomplish this, we exploited cognitively-plausible DSMs built upon non-spatial associative learning mechanisms to retrieve linguistic distances, while we quantified temporal distances by measuring the minimum travel time required to reach different Italian cities, selecting the main stations of Milan (Experiment 1A) and Rome (Experiment 1B) as departure points. The results of Experiment 1 revealed significant correlations between spatial, linguistic and temporal distances, highlighting the interconnected nature of these dimensions. Specifically, both Experiment 1A and Experiment 1B revealed a strong correlation between spatial and temporal distances, a moderate correlation between spatial and linguistic distances, as well as a moderate correlation between temporal and linguistic distances. Taken together, these findings support the notion that linguistic and temporal information can serve as proxies for estimating spatial relationships in large environments.

Building upon these findings, in Experiment 2 we explored whether linguistic and temporal information can explain human performance over and above spatial information. In particular, participants were required to identify which city, among each pair presented, was spatially closer to a referent point (i.e., Milan). Across both accuracy and response latencies, the results of Experiment 2 showed that, although spatial information played a major role in explaining participants’ performance, the effect of linguistic information was as well significant. By contrast, the temporal information did not yield significant effects. More precisely, results revealed that the more geographically and linguistically closer to Milan was one of the two cities composing the pairs (as compared with the other), the higher participants’ accuracy and the faster their response latencies. These findings indicate that linguistic information can account for human performance over and above the effect of spatial information (i.e., the model including linguistic information explains more variance than the model with the spatial predictor only) and thus that participants relied not only on spatial, but also on linguistic knowledge to solve the (space-centered) task. The current findings provide support to theoretical views sustaining the joint contribution of spatial and linguistic information in shaping and encoding the mental representations of maps representing large-scale environments (Louwerse, 2009, 2011, 2018; Louwerse & Jeuniaux, 2010; Friedman & Brown, 2000; Gatti et al., 2022; Tillman et al., 2013).

Importantly, these results provide support and extend previous evidence highlighting the key role played by linguistic experience in humans’ spatial cognition (Gatti et al., 2022; Tillman et al., 2013). A previous work by Tillman and colleagues (2013), indeed, reported analogous findings at the chronometric level but employed a task in which it was not possible to score participants’ accuracy, potentially introducing confounds in their observed effects. Additionally, their task involved specific iconic or reverse-iconic spatial arrangements of cities, which may limit the generalizability of their results. In contrast, our study employed a paradigm that allowed for scoring participants’ accuracy and did not imply any specific spatial arrangements. As a result, our findings provide a more comprehensive evaluation of the influence of linguistic experience on humans’ cognitive maps, suggesting a broader contribution of linguistic experiences than previously thought. Adding to this, it is interesting to note that evidence from a previous study by Gatti and collegues (2022) showed that participants’ chronometric performance in a task tapping on the judgment of absolute spatial locations was better predicted by language-derived coordinates (i.e., the location of a city with respect of the concepts of *North* and *South*) rather than by the real geographical coordinates. Taken together, this indicates that language contributes not only to the processing of absolute spatial information (i.e., location defined by coordinate axes), but also to distance-like information (i.e., location connected by paths but without specific reference to their orientation), further corroborating previous evidence demonstrating the successful reconstruction of geographical maps’ spatial layout from linguistic data (e.g., Louwerse & Zwaan, 2009; Louwerse & Benesh, 2012). Overall, these findings demonstrate not only that spatial information can be bootstrapped from natural language, but also that knowledge gained from purely linguistic data is not redundant in nature, but provide singular contributions in the organization and incorporation of distortions in spatial representation.

Notably, temporal information did not yield significant effects in the behavioral experiment. On the one hand, this lack of effects could be explained in light of the theoretical perspective of the metaphorical structuring, which states that space is the dominant concept influencing time perception, with time having a minimal impact on our perception of space (Boroditsky, 2000; Casasanto & Boroditsky, 2008; Lakoff & Johnson, 1980). The foundation of this theory is based on a linguistic ground, specifically, from the observation that human languages often employ a variety of spatial concepts to describe metaphorically temporal experiences and events (e.g., “We are approaching the deadline”). Taking an evolutionary perspective, the development of complex languages possibly prioritized the exchange of spatial information (e.g., “Which path led me to the food?”) over temporal ones (e.g., “At what time did I find it?”). Consequently, the use of spatial metaphors to describe temporal information could be attributed to the sequential integration of spatial and temporal concepts within language (Riemer et al., 2018; Srinivasan & Carey, 2010). Indeed, most languages exhibit a greater prevalence of metaphorical expressions that relate time to space compared to the reverse (Sinha & Gärdenfors, 2014). Additionally, a metaphorical link between time and space is also evident in gestures, as both children and adults often employ hand movement to describe temporal information as if they were physical location. For example, when indicating the past, people may gesture backwards, as if retracing steps along a path (Burns et al., 2019). However, it is also worth considering that while we quantified temporal distance as the minimum time needed to travel by train across Italian cities, alternative measures of temporal information could offer insightful perspectives as well as potential variations in results and interpretations. Note nevertheless that our decision to use train travel time than other ways of transportation is based on the relatively standardized nature of train schedules. Other ways, such as travel by car, are susceptible to significant individual variations (such as driving speed preferences) that can affect the accuracy of the temporal estimates.

Interestingly, our study provides valuable insights that complement the findings discussed by Friedman and colleagues (Friedman & Brown, 2000; Friedman & Montello, 2006; Friedman et al., 2002) in challenging the notion of cognitive map development primarily relying on spatial computations. However, while the works by Friedman and colleagues emphasize the crucial role of inferences through plausible reasoning, here our results highlight that the distributional structure of language is a further key source of spatial knowledge. Inferential accounts indeed suggest that, when individuals have to express judgments and make decisions about complex domains for which their knowledge is limited, as might often be the case for geographical knowledge (Friedman & Montello, 2006), they could resort to their partial information and engage in inferential processes (Friedman & Brown, 2000; Friedman & Montello, 2006; McNamara, 1986). While on the one hand we agree that inferential processes might play a crucial role in performing geographical judgments and in structuring geographical knowledge, our findings also indicate a pivotal role of the (non-inferential) distributional history of words in natural language as a primary source of information. Notably, previous studies have shown that DSMs, despite their non-inferential architecture, exhibit the ability to generate reliable inferences about the world and its entities (e.g., Berlin: Germany = Rome: x, where x = Italy; see: Marelli, 2017). Consequently, our findings should not be interpreted as suggesting that inferential and associative-learning processes are mutually exclusive sources of cognitive map development and organization. Instead, we believe that the distributional history of words in natural language can be conceived as one of the fundamental bases for the development of higher-level cognitive inferential processes.

In summary, these findings contribute to ongoing discussions about the development and organization of cognitive maps in humans by providing a more comprehensive understanding of the factors that contribute to mental representations of maps representing large-scale environments. In particular, this is the first study exploring whether distortions encoded in cognitive maps can be explained in light of prior knowledge about both linguistic and temporal information, as assessed by participants’ accuracy and chronometric performance in a computerized task. Future research could apply the methods used here (as well as other recent computational models: e.g., Günther et al., 2023) to investigate the influence of spatial, linguistic and temporal factors on cognitive mapping across different environmental scales, including small (e.g., rooms or short pathways) and medium scale (e.g., neighborhoods or cities) environments, as well as investigating individual differences as potentially contributing factors in the development and organization of cognitive maps. That is, while within this topic a large body of evidence is available for temporal information (Clayton et al., 1991; Hanyu et al., 1995; MacEachren, 1980; McNamara et al., 1984, 1992), no evidence is available regarding linguistic information. The evidence presented here challenges prevailing views aligned with embodied accounts of cognition (Barsalou, 2008; Gallese & Lakoff, 2005) that prioritize specialized spatial computations (Bellmund et al., 2018; Bottini & Doeller, 2020a, b; Derdikman & Moser, 2010). In contrast, our findings provide support for theoretical positions that argue for the importance of non-spatial processes in shaping spatial knowledge (Louwerse, 2009, Louwerse, 2018; Friedman & Brown, 2000; Gatti et al., 2022; Shrager et al., 2008).

## Data Availability

A version of this manuscript has been uploaded on PsyArXiv (https://osf.io/preprints/psyarxiv/3a6tr) and the complete set of scripts and data is available at: https://osf.io/pexqm/. This study was not preregistered.
